# Supplementary material for: A Comprehensive Assessment of Ultraviolet-Radiation-Induced Mutations in Flammulina filiformis Using Whole-Genome Resequencing
Source: J Fungi (Basel). 2024 Mar 20;10(3):228. doi: 10.3390/jof10030228 (PMC10971301; doi:10.3390/jof10030228)
Supplement: Supplementary file 1 [file jof-10-00228-s001.zip › Supplementary Material S8/KEGG annotation/out/64381550635650.os/KO/out_map/map04726.html]

KEGG PATHWAY: Serotonergic synapse - Reference pathway


|  |  |
| --- | --- |
| **Serotonergic synapse - Reference pathway** |  |

[
Pathway menu
| Organism menu
| Pathway entry
| Show description
| User data mapping
]

Serotonin (5-Hydroxytryptamine, 5-HT) is a monoamine neurotransmitter that plays important roles in physiological functions such as learning and memory, emotion, sleep, pain, motor function and endocrine secretion, as well as in pathological states including abnormal mood and cognition. Once released from presynaptic axonal terminals, 5-HT binds to receptors, which have been divided into 7 subfamilies on the basis of conserved structures and signaling mechanisms. These families include the ionotropic 5-HT3 receptors and G-protein-coupled 5-HT receptors, the 5-HT1 (Gi /Go -coupled), 5-HT2(Gq-coupled), 5-HT4/6/7 (Gs-coupled) and 5-HT5 receptors. Presynaptically localized 5-HT1B receptors are thought to be the autoreceptors that suppress excess 5-HT release. 5-HT's actions are terminated by transporter- mediated reuptake into neurons, leading to catabolism by monoamine oxidase.

|  |  |
| --- | --- |
| Reference pathway | 100% |
